# Conditioned Medium of Adipose-Derived Mesenchymal Stem Cells as a Promising Candidate to Protect High Glucose-Induced Injury in Cultured C28I2 Chondrocytes

**DOI:** 10.34172/apb.2022.044

**Published:** 2021-08-15

**Authors:** Sedighe Safari, Akram Eidi, Mehrnaz Mehrabani, Mohammad Javad Fatemi, Ali Mohammad Sharifi

**Affiliations:** ^1^Department of Biology, Science and Research Branch, Islamic Azad University, Tehran, Iran.; ^2^Physiology Research Center, Institute of Neuropharmacology, Kerman University of Medical Sciences, Kerman, Iran.; ^3^Burn Research Center, Motahari Hospital, Iran University of Medical Sciences, Tehran, Iran.; ^4^Department of Pharmacology, School of Medicine, Iran University of Medical Sciences, Tehran, Iran.; ^5^Stem Cell and Regenerative Medicine Research Center, Iran University of Medical Sciences, Tehran, Iran.; ^6^Tissue Engineering Group, (NOCERAL), Department of Orthopedics Surgery, Faculty of Medicine, University of Malaya, Kuala Lumpur, Malaysia.

**Keywords:** Adipose derived mesenchymal stem cells, Conditioned medium, High glucose, Oxidative stress

## Abstract

***Purpose:*** The aim of this study was to evaluate the protective effect of conditioned medium derived from human adipose mesenchymal stem cells (CM-hADSCs) on C28I2 chondrocytes against oxidative stress and mitochondrial apoptosis induced by high glucose (HG).

***Methods:*** C28I2 cells were pre-treated with CM-hADSCs for 24 hours followed by HG exposure (75 mM) for 48 hours. MTT assay was used to assess the cell viability. *Reactive oxygen species* (ROS) and lipid peroxidation were determined by 2,7-dichlorofluorescein diacetate (DCFHDA) and thiobarbituric acid reactive substances (TBARS) assays, respectively. Expressions of glutathione peroxidase 3 *(GPX 3), heme oxygenase-1 (HO-1),* and NAD(P)H quinone dehydrogenase 1 (*NQO1*) were analyzed by RT-PCR. Finally, western blot analysis was used to measure Bax, Bcl-2, cleaved caspase-3, and Nrf-2 expression at protein levels.

***Results:*** CM-hADSCs pretreatment mitigated the cytotoxic effect of HG on C28I2 viability. Treatment also markedly reduced the levels of ROS, lipid peroxidation, and augmented the expression of *HO-1, NQO1,* and *GPx3* genes in HG-exposed group. CM-ADSCs enhanced Nrf-2 protein expression and reduced mitochondrial apoptosis through reducing Bax/Bcl-2 ratio and Caspase-3 activation.

***Conclusion:*** MSCs, probably through its paracrine effects, declined the deleterious effect of HG on chondrocytes. Hence, therapies based on MSCs secretomes appear to be a promising therapeutic approaches to prevent joint complications in diabetic patients.

## Introduction

Diabetes mellitus (DM) is a common degenerative disease characterized by chronic elevated levels of blood glucose or hyperglycemia due to insufficient or inefficient insulin secretory response.^1^ It seems that mono-target therapy fails to completely manage diseases with multifactorial pathogenesis such as DM. Hence, therapies focusing on multi-targets such as stem cell-based therapies received much attention in the recent years.^2^ DM adversely influences various cell types, thereby increases the risk of development and progression of other diseases such as osteoarthritis (OA).^3,4^ OA is a slowly progressive joint disorder of aged people manifested by destruction of articular cartilage.^5^ Chondrocytes of articular cartilage are responsible for maintaining cartilage homeostasis.^6^ However, many factors including HG condition as the most hallmark of DM may cause chondrocyte dysfunction and OA onset.^7,8^ Normally, glucose plays an important role in the metabolism of chondrocytes. However, high amount of blood glucose in diabetic condition can saturate the glycolytic pathway leading to the entrance of glucose to other alternative glucose metabolic pathways and induction of oxidative stress.^9^ Oxidative stress activates several signaling pathway involved in inflammation, dysregulation of the glutathione antioxidant system and activating intrinsic or extrinsic apoptosis pathway.^10^ It is well documented that oxidative stress and apoptosis are crucial events in the pathogenesis of OA.^8,11^

Nuclear factor (erythroid-derived 2)-like 2 (Nrf2) is considered one of the major cellular defense mechanism against oxidative stress.^12^ It plays an important role to protect tissues and cellular components from ROS-induced oxidative damage by regulating expression of antioxidant and cytoprotective enzymes including Heme oxygenase-1 (HO-1), NAD(P)H dehydrogenase [quinone]1(NQO1), superoxide dismutase (SOD), glutathione peroxidase (GPx) and catalase (CAT).^13^

Current pharmacological therapy often focused on the relieving from the OA-related symptoms and/or controlling the inflammation process.^14^ Furthermore, surgical treatment to repair the damaged cartilage could only decreases pain for a short time.^15^ It seems that the most of the current therapies for OA have little long-term benefits. Hence, efforts have been ongoing to find more efficient approaches to combat the progression rate of OA. Currently, mesenchymal stem cells (MSCs) has attracted great attentions as a promising therapeutic option for different diseases including OA.^16-18^ MSCs are multipotent stem cells derived from different tissues including bone marrow, adipose, and other tissues. The ease of isolation and expansion and high potency of multi-lineage differentiation have made the MSCs the most encouraging candidate for the cartilage tissue engineering.^19^ Nonetheless, the clinical use of MSCs still remains challenging. For example, MSCs may have pro-tumorigenic activity, elicit an immune response, differentiate into undesirable tissue, or show low survival after transplantation.^20^ Hence, cell free therapeutic approaches such as MSC-conditioned medium (MSCs-CM) therapies would be more affordable, controllable and practical.^21^ A growing evidence have demonstrated that the beneficial impacts of MSCs would be mediated via paracrine mechanisms.^22^ Research illustrated the secretion of numerous bio-active factors such as cytokines, growth factors, microRNA, proteasomes, as well as exosomes by MSCs.^23,24^ It was shown that conditioned medium derived from Wharton’s jelly derived stem cell can improve the gene expression profile of collagen II, sox-9, cartilage oligomeric matrix protein and aggrecan by chondrocytes.^25^ Furthermore, conditioned medium from adipose-tissue-derived mesenchymal stem cells exerted a chondroprotective effect probably by inhibition of nuclear factor-*κ*B activation.^26^ A previous investigation reflected that CM from the MSCs could modulate immune responses in uveitis.^27^ Regarding the therapeutic benefits of cell free products of MSCs, the present study aimed to investigate the protective effect of MSCs-CM against the high glucose-mediated oxidative stress as well as consequent apoptosis in the C28I2 human chondrocytes.

## Materials and Methods

### Antibodies and reagents

Dulbecco’s Modified Eagle’s Medium/Nutrient F-12Ham, penicillin–streptomycin, amphotericin B, and fetal bovine serum (FBS) were purchased from Gibco (Invitrogen, Carlsbad, CA). Moreover, TRIzol reagent was obtained from Invitrogen (Merelbeke, Belgium). The antibodies were from the Abcam (UAS). Polyvinylidene fluoride (PVDF) membrane was from Bio-Rad (Hercules: CA) and enhanced chemiluminescence (ECL) kit was from Amersham Bio-sciences (Buckinghamshire: UK). All materials for differentiation assay,phosphate-buffered saline (PBS), 3-(4,5-dimethylthiazol-2-yl)-2,5-diphenyltetrazolium bromide** (**MTT), NaCl, KCl, HEPES, EDTA, and DMSO were provided from Sigma Co. (Sigma Aldrich, St Louis, MO). Fluorescein isothiocyanate (FITC)- conjugated mouse anti-human against CD105, CD90, CD44, CD31, CD34, and HLA-DR were purchased from eBioscience (USA).

### C28I2 culture

The chondrocyte cell line, C28I2, was provided from Pasteur Institute (Tehran, Iran). The growth medium of C28I2 contained DMEM/F12 complemented with 10% FBS as well as 1% (v/v) penicillin–streptomycin (100 U/mL penicillin & 100 µg/mL streptomycin).

### Human adipose-derived stem cells (hADSCs) isolation and culture

Adipose tissues were obtained from five healthy donors without any underlying diseases after elective abdominal liposuction at the surgical center of Milad Hospital, Tehran, Iran. For isolation of hADSCs, after washing with PBS, adipose tissue was exposed to 0.075 % collagenase type Ι (Sigma Aldrich; St Louis; MO: USA) for 30 minutes in 37°C with moderate agitation. Afterwards, centrifugation has been done at 600 g for 5 minutes. In the next step, the pellets were incubated with NH4Cl on ice for five minutes. After that, cell pellets have been resuspended in 5 mL α-MEM consisting of FBS 20%, penicillin/streptomycin 1%, L-glutamine (Gibco, Invitrogen) 1 % and Fungizone 0.1 %. The cell suspension was filtered through a 100 µm nylon mesh to remove debris. The filtrated samples were cultured into a tissue culture flasks and incubation has been done at 37ºC-, 5% CO2.^28^

### Characterization of hADSCs

#### Flow cytometry

Expressions of hADSCs surface markers were determined by fluorescence-activated cell sorting (FACS) analysis using a BD FACSCalibur flow cytometer (Becton Dickinson, San Diego, CA, USA). In brief, the hADSCs at 3-4 passages were trypsinized and then centrifuged at 2000 rpm for five minutes. Afterwards, cells have been resuspended in PBS and 2% FBS. Then, 100 µL of cell suspension has been incubated with 5 µL of antibodies including: IgG1-FITC-Isotype control, IgG1-PE-Isotype control, PE- Conjugated anti-CD44, PE- conjugated anti-CD105, PE-Conjugated HLA-DR, APC-conjugated anti-CD90, FITC-conjugated anti-CD34 antibody and *PE/Cy5* -conjugated CD45 for 40 minutes on ice in darkness. At the end, the outputs have been analyzed with the Cell Quest software.

### Differentiation assay

#### Adipogenic differentiation

To prove the adipogenic potential of the hADSCs, cells have been incubated in the 6-well plate until 80% confluent. Then, we added the adipogenic induction medium consisting of α-MEM with 10% FBS, 100 µg/mL streptomycin, 100 U/mL penicillin, 10 μg/mL insulin, 2mM L-glutamine, 1 μM dexamethasone, 500 μM IBMX, and 100 μM indomethacin. Following 14 days, these cells have been fixed with 10% formalin and finally 0.5 % Oil Red O has been used to stain the cells.

### Osteogenic differentiation

To demonstrate the osteogenic potential of hADSCs, cells were plated until they became fully confluent and then osteogenic induction medium was added, consisting of α-MEM complemented with penicillin (100 U/mL), 10 % FBS, streptomycin (100 μg/mL), L-glutamine (2mM), dexamethasone (100 nM), ascorbic acid (50 μM), and β-glycerol phosphate (10mM). The medium was changed every three days. On day 14, cells have been stained with 10% Alizarin Red.

### Chondrogenic differentiation

For demonstration of the chondrogenic potential of hADSCs, cells at density of 5×10^5^ has been pelleted in the 15 ml polypropylene conical tubes. Then, incubation of the cells has been performed at 37°C, 5% CO2 with complete chondrogenic medium, using The StemPro® Chondrogenesis Differentiation Kit. This medium has been replaced 3 times a week. On day 21, we harvested the pellets, placed them in paraffin, and consequently sectioned them (5 μm thickness). Finally, Alcian Blue Solution has been used to stain the pellets.

### Collection of conditioned media

hADSCs at 80-90% confluence were washed three times with the PBS and consequently the medium was replaced with the serum-free α-MEM consisting of 1% penicillin-streptomycin. CM has been collected after 48 hours of culture. Then, centrifugation of CM has been done at 1500 rpm for five minutes for the removal of the debris. Finally, CM has been stored at -80˚C until the day of experiment.

### MTT assay

C28I2 cellswere plated into 96-wells at a density of 10^4^. After 24 hours, cells were washed three times with serum-free media and incubated with CM-hADSCs or serum-free media (50/50 v/v%) based on the previous studies.^29,30^ One day later, the cells have been exposed to HG (75 mM) for 48 hours.^31^ The protective effect CM was investigated using MTT assay. MTT solution has been added to the wells and thus incubation of the plates has been performed at 37°C for four hours. At the next step, the medium has been removed and 100 μL dimethyl-sulfoxide (DMSO) has been added into all wells. Finally, we used a microplate reader (Bio-Tek ELX800; Winooski, VT, USA) to measure the solution absorbance at 570 nm.^32^

### Measurement of intracellular reactive oxygen species (ROS)

Briefly, the C28I2 cells were pre-treated with or without CM- hADSCs/serum free media (50/50 v/v%) for 24 hours followed by exposing to the HG (75 mM) for 48 hours. After removing the media and washing with PBS, cells were incubated with 2,7-dichloro-fluorescein diacetate (DCF-DA) for 30 minutes. Furthermore, 485 nm excitation and 528 nm emission wave-length have been used to measure intracellular ROS activities with a Synergy HT Microplate Reader (BioTek ELx800; Winooski, VT, USA).

### Thiobarbituric acid reactive substances (TBARS) assay

The lipid peroxidation level has been measured through TBARS assay as described previously.^33^ Briefly, after homogenization, centrifugation of the homogenate has been done at 1000×g for 10 minutes. The supernatant has been added to thiobarbituric acid (0.8%), sodium dodecyl sulphate (8%), and acetic acid (20%). Each sample has been heated at 95°C for sixty minutes and cooling has been done slowly to the room temperature. In the next step, n-butanol has been added. Following centrifuging, the absorbance of the organic layer was measured at 532 nm.

### Reverse transcription-polymerase chain reaction (RT-PCR)

Total RNA has been extracted using the TRIzol reagent (Invitrogen Co., Carlsbad, CA, USA) according to the Company’s directions. For synthesizing cDNA, RNA was mixed with M-MLV RT, oligo dT, RNase Inhibitor, and deoxyribonucleotide triphosphate (dNTP) in a final volume of 20 mL. The reaction was performed at 42°C for one hour and 72°C for 10 minutes. Then, we exposed cDNA to PCR. After that, it has been amplified in an Eppendorf Master cycler (Hamburg: Germany). Furthermore, β-actin has been utilized as an internal control. Finally, PCR products were run on the 2% agarose electrophoresis gel and the red-safe staining has been chosen to visulaize them. Table 1 displays the applied primers.

**Table 1:** Rat primers for RT‐PCR

**Gene**	**Sense primer(5΄→3΄)**	**Anti-sense (5΄→3΄)**	**Size (bp)**
HO-1	GCTGAC CCA TGA CAC CAA G	GTG TAA GGA CCC ATC GGA GA	161
NQO1	ACT GAT CGT ACT GGC TCA CTC	CCT TCA GTT TAC CTG TGA TGT CC	167
GPx3	CAC GAC ATC CGC TGG AAC TT	AGT CCC TCC CCT ACA TGG TG	197
β-actin	CAC CAT GGA TGA TGA TAT CGC	TGG ACG ATA GCT TGG AGG GA	467

### Western blot

Briefly, the treated and non-treated cells were lysed by RIPA lysis buffer, phosphatase inhibitor cocktails, and proteases. Afterwards, centrifugation was performed at 12000×g at 4°C for 30 minutes. Protein concentration has been measured by Bradford assay.^34^ The SDS–PAGE polyacrylamide gel (10%) was used to separate the samples of the proteins at 150 V for one hour. In the next step, the proteins were transferred to the membrane PVDF. Next, the primary antibodies (Bax, Bcl-2, cleaved caspase-3, Nrf2 and β-actin) and then the horseradish peroxidase labeled secondary antibody have been utilized to incubate a PVDF membrane. Consequently, the ECL detection reagents have been utilized for visualization of the bands. Finally, ImageJ software has been applied for the quantification of the Band density.^35^

### Statistical analyses

For analyzing of the statistical differences between two independent groups or multiple groups, un-paired student’s *t*test and one-way ANOVA with Tukey’s test were used respectively. Data were stated as mean ± SEM. The statistical values at *P* < 0.05 have been set to be significant.

## Results and Discussion

### hADSCs characterization

**Figure 1:**
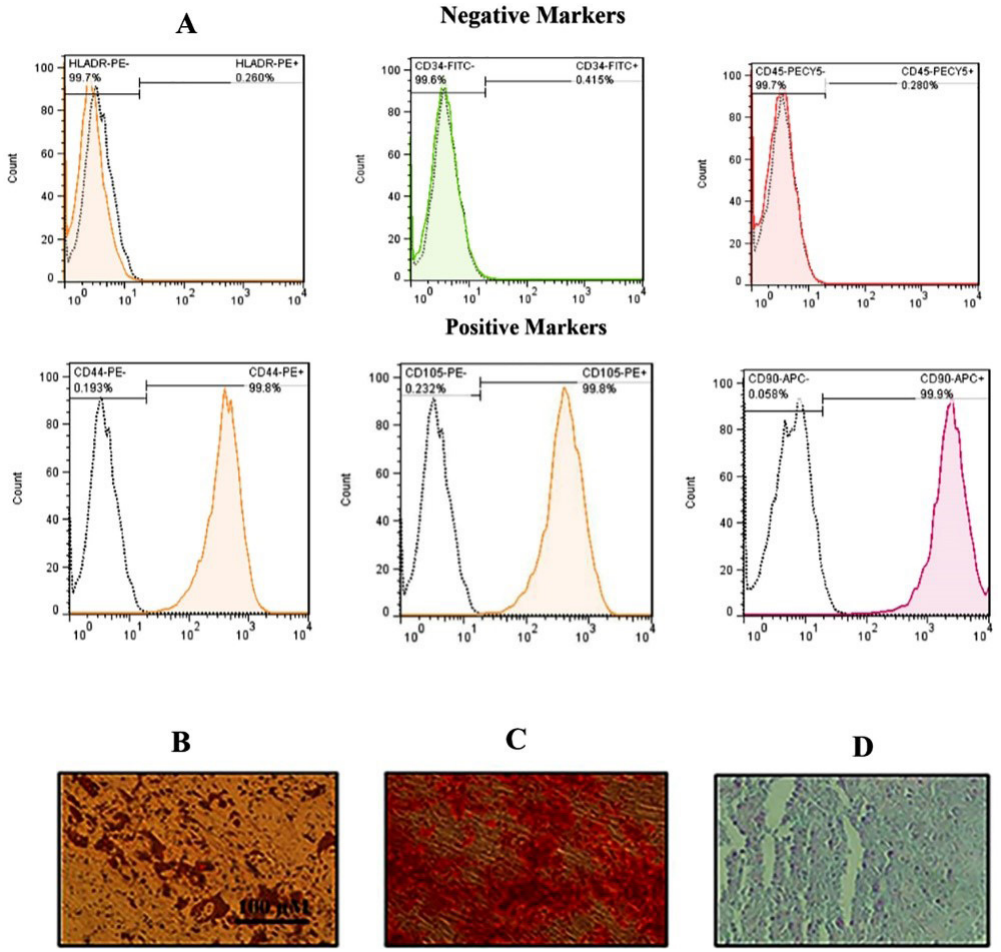
Flow cytometry histograms and differentiation images for hADSCs. (A) Flow cytometry illustrated that hADSCs displayed > 95% positivity for CD90, 105 and CD 44 and < 0.5% positivity for HLA-DR and CD34 as well as CD45. Unstained cells were used as a control. Differentiation images of the hADSCs into the adipocytes (Oil red staining) (B), osteoblasts (Alizarin red staining) (C) and finally chondrocyte (Alcian blue staining) (D) evaluated under light microscopy.

The characterization of hADSCs was confirmed by flow cytometry. Results showed that the hADSCs (passages 3 to 4) have been positive for CD44, CD90, and CD105; however, they have been negative for CD34, CD45, and HLA-DR markers (Figure 1A). Alizarin Red staining and Oil Red O revealed that cells were stained positive for the lipid vacuoles (Figure 1B) as well as the calcium deposits (Figure 1C), which verified the cells’s adipogenic and chondrogenic differentiation, respectively. Moreover, alcian blue staining indicated glyco-saminoglycans in the chondrocyte following 21 days (Figure 1D).

### Effect of CM-hADSCs on the viability of C28I2 cells exposed to HG condition

**Figure 2:**
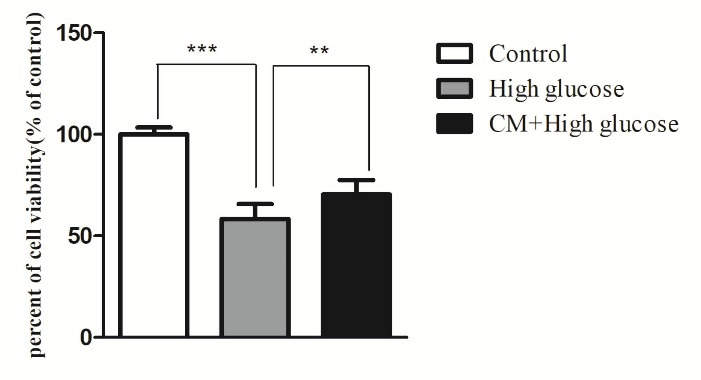
Protective effect of CM-hADSCs on the viability of C28I2 cells against HG conditions. Cell viability was assessed by MTT colorimetric assay. HG decreased C28I2 cells viability and CM pretreatment could significantly reverse its effect. Values are mean ± standard error of the mean (SEM). ***P < 0.001, ** *P* < 0.01 versus (vs.) high glucose (HG) (n = 8).

The current research has investigated the protective effect of CM-hADSCs against HG-induced damage in C28I2 cells and the possible underlying mechanisms. Pretreatment of C28I2 cells with CM-hADSCs reduced the negative effect of HG on the viability of HG-exposed C28I2 cells.As shown in Figure 2, HG (75 mM) markedly decreased viability of C28I2 cells after 48 hours.Similar to the current study, a previous work showed that HG (75 mM) treatment for 48 hours could significantly decrease C28I2 chondrocyte viability compared to the control group.^36^ However, pretreatment of C28I2 cellswith CM-hADSCs (50%) for 24 hours significantly inhibited the deleterious effect of HG on the viability of C28I2 cells. Some previous studies have shown the protective effect of CM at 50% concentration on different cell types.^29,30^ Another study has also demonstrated that CM derived from human umbilical cord–derived mesenchymal stromal cells was able to increase the viability of cardiac fibroblast cells against irradiation-induced damage. Such positive effects may be due to the presence of many growth factors, chemokines, and hormones in stem cell-derived CM.^37^

### Effect of CM-hADSCs on the intra-cellular ROS generation and TBARS levels of C28I2 cells exposed to HG condition

**Figure 3:**
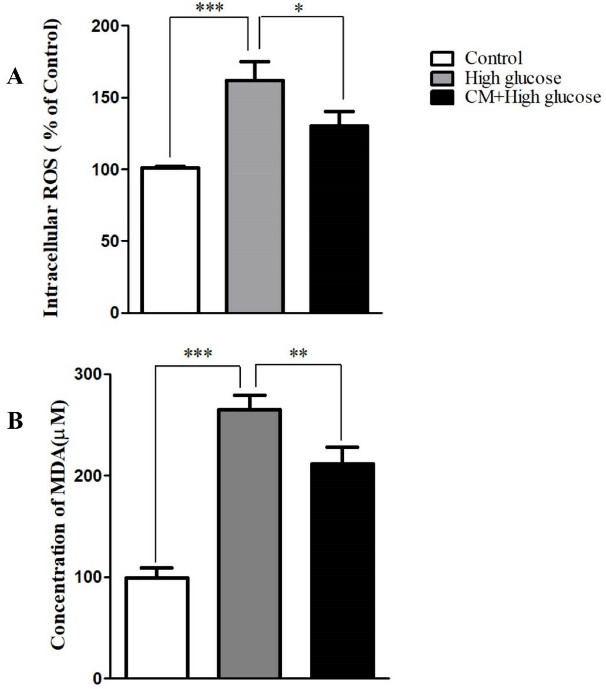
The effects of CM-hADSCs on ROS formation and MDA level of the C28I2-cells exposed to HG condition. (A) Intracellular accumulation of the ROS increased by HG (75 mM) exposure whereas the CM-hADSCs pre-treatment significantly decreased ROS formation, (B) lipid per-oxidation was augmented in the HG-treated group. CM-hADSCs reduced the HG-induced lipid per-oxidation in the C28I2-cells. Values are presented as mean ± SEM.; * *P* < 0.05, ** *P* < 0.01, ***P < 0.001 vs. HG (n = 3).

As shown in Figure 3A and B, the intra-cellular ROS and TBARS levels were significantly augmented following exposure to HG (75 mM) for 48 hours in comparison with the control group. However, pretreatment of C28I2 cellswith CM-hADSCs (50%) for 24 hours significantly declined the effects of HG on ROS production and TBARS level. HG induces oxidative stress by different mechanisms for instances NADH production and increasing the advanced glycation end products.^38^ It was also shown that chondrocyte in response to HG environment generated the high level of ROS.^9^ ROS and reactive nitrogen species are involved in degrading cartilages and causing chondrocyte senescence by direct attack and/or indirect activation of the latent matrix degrading enzymes, preventing the synthesis of the cartilage matrix macro-molecules and induction of apoptosis.^39,40^ Growing evidences have shown that CM of MSCs have potential antioxidant activity. For example, CM derived from MSCS significantly decreased MDA and ROS levels, and increased GSH activity in a model of ureteral obstruction‐induced kidney injury.^41^ Furthermore, ADSCs-CM was shown to exert a potent antioxidant activity against oxidative stress induced by tert-butyl hydroperoxide in fibroblast cells.^42^

### Effect of CM-hADSCs on the expression of Bcl-2 and Bax protein levels and on the caspase-3 activation of C28I2 cells exposed to HG condition

**Figure 4:**
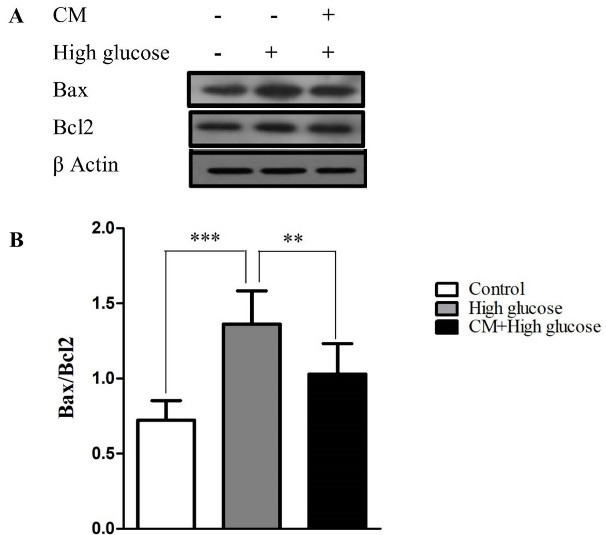
The effects of CM-hADSCs on the protein expression ratio of Bax/Bcl-2 in the C28I2 cells exposed to HG condition. (A) Representative blot and (B) and semi‐quantitative data of protein expression of Bax and Bcl2 protein in the C28I2 cells. The ratio of Bax/Bcl-2 increased in the HG-treated group whereas the CM pre-treatment considerably diminished the effect of HG. Values are presented as mean ± SEM. ***P < 0.001, ** *P* < 0.01 vs. HG (n = 3).

**Figure 5:**
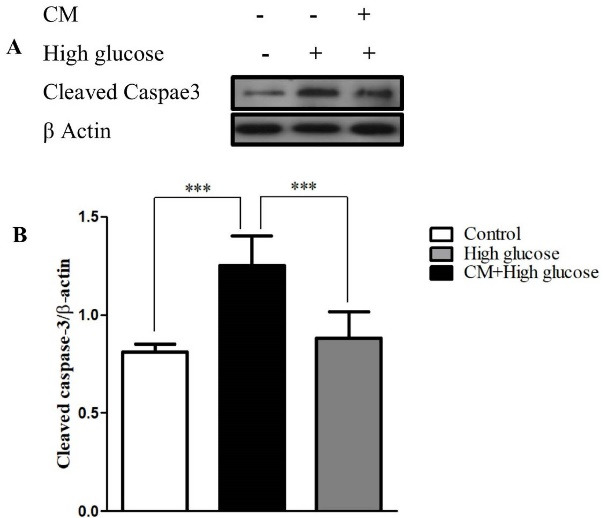
The effects of CM-hADSCs on the protein expression of cleaved caspase-3 in the C28I2 cells exposed to HG condition. (A) Representative blot (B) and semi‐quantitative analysis of cleaved caspase-3 at protein level in the C28I2 cells. As seen, the cleaved caspase-3 enhanced in the HG-treated group and CM pretreatment declined HG effect. Values are presented as mean ± SEM. ***P < 0.001 vs. HG (n = 3).

As shown in Figure 4, the HG enhanced the ratio of Bax/Bcl2 in C28I2 cellsas compared to the controls. The pretreatment of C28I2 cellswith CM-hADSCs (50 %) for 24 hours prior to adding HG reversed the effect of HG on Bax/Bcl-2 ratio. Results showed that HG augmented the cleaved caspase-3 protein level in comparison with the control group. The pretreatment of C28I2 cellswith CM-hADSCs (50%) for 24 hours decreased the level of the cleaved caspase-3 protein in C28I2-cells (Figure 5). Apoptosis happens through activating a group of cysteine proteases termed caspases including initiator caspases (caspases 2, 8, 9, & 10) and effector caspases (caspases 3, 6, & 7). The antiapoptotic Bcl-2 family members like Bcl-2 can prevent releasing of cytochrome c from the space of the inner membrane of mitochondria. However, apoptosis progresses when proapoptotic proteins like Bax localized in mitochondrial membranes where they enhance membrane permeability. Eventually, the released cytochrome c leads to activation of caspase-3 as well as capase-9 which induce apoptosis.^43-45^ HG can trigger apoptosis in chondrocytes by elevating Bax/Bcl-2 ratio, caspase-3 activation, and JNK and P38 phosphorylation.^31^ In OA cartilage, the expression of Bcl-2 is lower and caspases-3 is higher than healthy cartilages, and it was demonstrated that inhibition of caspases was capable to improve chondrocytes viability and function.^46,47^ Thus, suppressing subcellular signaling pathways regulating chondrocyte apoptosis and articular cartilage degradation may offer a potential target for therapeutic purposes in OA cartilage. These data were confirmed by a pervious study showing that MSC-derived CM demonstrated abilities to promote the survival and decrease apoptosis in the 2,5-hexanedione-induced apoptosis in PC12-cells via inhibiting mitochondrial pathway.^48^ Another study by Tarng et al showed that protective factors in CM could be utilized as an antioxidant, antiapoptotic and anti-inflammatory factors in kidney diseases.^49^

### Effects of CM-hADSCs on the expression of Nrf2

**Figure 6:**
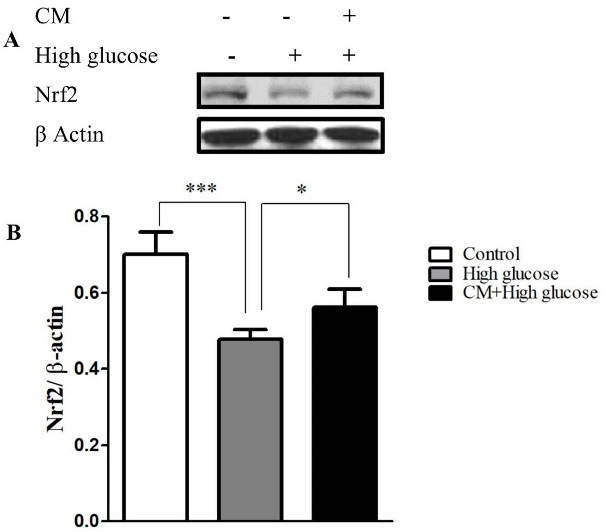
The effects of CM-hADSCs on the protein expression of Nrf2 in the C28I2 cells exposed to HG condition. (A) Representative blot (B) and semi‐quantitative analysis of NRF-2 at protein level in the C28I2 cells. Nuclear Nrf2 was elevated in the CM pre-treated groups in comparison with HG-group. Values are presented as mean ± SEM. *P < 0.05, ***P < 0.001 vs. HG group (n = 3).

Nrf2 is a major cellular defense mechanism against oxidative stress. CM-hADSCs improved Nrf-2 protein expression and reduced mitochondrial apoptosis. As shown in Figure 6, HG could significantly reduce the expression of Nrf2 in comparison with the control group, while pretreating with CM-hADSCs for 24 hours considerably enhanced the level of Nrf2 protein as compared with the HG group. One possible mechanism involved in the antioxidant activity of stem cells is the activation of Nrf-2. Nrf2 has been considered as one of the transcription factors, which regulates genes associated with the anti-oxidant system of the cells. In normal condition, Nrf2 would be maintained in the cytoplasm by attaching to the Kelch-like ECH-associated protein 1 (Keap1). Therefore, in response to the mild oxidative stress, Nrf2 is released from the keap1 and translocated into nucleus.^50,51^ However, high doses of ROS could decrease Nrf-2 expression.^52,53^

### Effects of CM-hADSCs on the anti-oxidant enzymes at mRNA level

**Figure 7:**
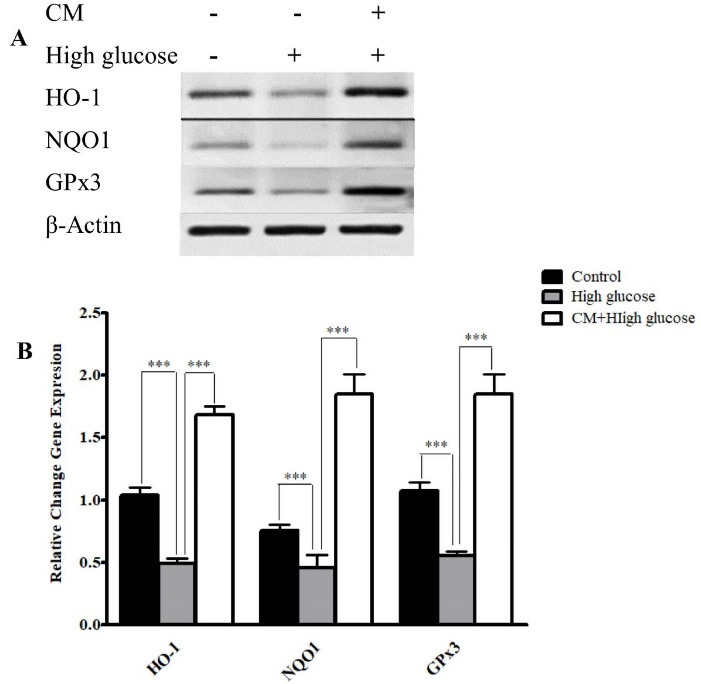
The effects of CM-hADSCs on the mRNA expressions of NQO1, GPx3, and HO-1 in the C28I2 cells exposed to HG condition. (A) Representative image (B) and semi‐quantitative analysis of NQO1, Ho-1, and GPx3 at mRNA level in the C28I2 cells. RT-PCR analysis has shown that HG decreased the expression of NQO1, Ho-1, and GPx3 at mRNA levels in comparison with the control. Pre-treatment with CM could considerably augmented HO-1, NQO1 and GPx3 expression in comparison with the HG group. Values are presented as mean ± SEM. ***P < 0.001 vs. HG group (n = 3).

The expression of mRNA of *HO-1*, *NQO1*, and *GPx3* significantly decreased following treatment with HG for 48 hours as compared to the controls. The results showed that CM-hADSCs could increase mRNA expression of target genes of NRF-2 such as *NQO1*, *HO1* and *GPx* (Figure 7). A previous study showed that CM of human placental MSCs could activate Nrf-2 signaling pathway and increase the expression of HO-1, thereby protected the cells from oxidation-induced apoptosis.^51^ Furthermore, MSC-CM therapy improved the vascular injury in DM via the regulation of ROS levels and decreasing the oxidative injury via increasing of antioxidant enzyme activity, including CAT and SOD.^54^ Another report showed that advantageous of CM could be associated with its antioxidant capacity and the presence of growth factors and activation of Nrf2.^55^ Nrf2 via activation of ERK1/2/ELK1-P70S6K-P90RSK signaling axis had anti-oxidative and anti-apoptotic function in in IL-1β stimulated OA chondrocytes.^56^ It was also revealed that NRF-2, by increasing HO-1, decreased HG-induced expression of caspase 3 and cleaved caspase3 in HK-2 cells.^57^

## Conclusion

The current results demonstrated that the pretreatment of C28I2 chondrocytes with CM-hADSCs could decrease the deleterious effect of HG probably by augmentation of the antioxidant capacity of cells and attenuation of mitochondrial apoptosis. Hence, CM-hADSCs may consider a promising treatment modality in treating patients with progressive destruction of articular cartilage as a complication of DM.

## Acknowledgments

The authors are acknowledged Mrs. Maroof for her support. The present study has been supported by a grant from National Institute of Medical Research and Development (NIMAD).

## Ethical Issues

Not applicable.

## Conflict of Interest

The authors declare no conflict of interest.
